# Hopital Saint Antoine

**Published:** 1830-07

**Authors:** 


### HOPITAL SAINT ANTOINE.

### *Use of Oil of Turpentine in Neuralgia.* By M. Rayer.

Since the appearance of M. Martinet's observations on the employment of oil of turpentine in cases of neuralgia, and especially in sciatica, various practitioners have had recourse to this remedy with success. The following cases show the utility of turpentine in these always painful, and too frequently perplexing maladies.

Case I. A man, æt. sixty-six, was admitted, in May 1829, with the ordinary symptoms of intense facial neuralgia of the right side. The disease had existed for twelve years, and it first appeared after the suppression of a rheumatic affection of the right arm, which had lasted fifteen months. The patient had been in several hospitals, but, in spite of the treatment that had been practised by different eminent physicians, the lancinating pains in the face were not relieved. Leeches and blisters to the cheek, general bleeding, acupuncturation, the extraction of four teeth, valerian, extract of belladonna, had all proved of no avail. The facial nerve had been divided, but, of course, without any benefit.

When the patient entered the hospital of St. Antoine, he was suffering the most dreadful torments. The pain was seated deep in the orbit, in the temporal fossa, in the superior alveolar process, and in the sub-orbitar region. Sometimes it darted over the whole of the right side of the face, while at other times it was confined to a well-marked line in the track of one of the branches of the affected nerve. It attacked by paroxysms, which lasted from one to ten minutes. Several paroxysms occurred during each day, and their frequency appeared to be increased when the patient had either eaten or spoken often. When the attack was very severe, the integuments under the eye were wrinkled, the muscles of the face were convulsively contracted, the secretion of tears very abundant, and sometimes the jaws were violently and suddenly closed. In this state the patient was neither capable of speaking, nor of attending to what was said to him. There was neither redness nor swelling of the face. Either a warm or humid temperature was much feared, but from dry and cold weather some relief was obtained.

M. Rayer advised the application of an opiate plaster, which was renewed several times a day, without any benefit.

June 2d.—He commenced with half a drachm of oil of turpentine in a mixture, and the dose was gradually increased to two drachms. Each day the relief was evident, and on the twelfth from the commencement of the remedy the pains were considerably diminished, and the attacks much less frequent.

15th.—As the stomach and bowels appeared to be irritated by the turpentine, it was omitted, and tartar-emetic ointment was directed to be rubbed in upon the cheek for one week.

25th.—The attacks have been repeated with increased violence. The oil of turpentine was again given, and in five days each dose was one drachm and a half. The patient was again decidedly relieved, but, as his stomach would not bear the remedy, it was unwillingly discontinued; and during the following days half a grain of tartar emetic was occasionally given in pills, which produced vomiting.

July 15th.—The patient was anxious to leave the hospital, as he was so much better. He had now but three or four slight attacks in forty-eight hours, and when he was admitted he had twenty-five or thirty very violent paroxysms in the same space of time.

On the 17th August, he was again admitted. The pains were now as frequent and severe as at first. Again the turpentine was administered with much advantage, but, from the idiosyncrasy of the patient, it could not be continued. From various other remedies no further relief was obtained, and the man left the hospital to go into the country.

Case II. January 16th, a woman, ætat. forty-four, was admitted. She had been much exposed to bad weather, and, after a wandering rheumatic affection, was now labouring under very severe sciatica. During twelve days camphor pills were given, but, after a temporary relief, the pain returned with increased violence. She was bled to the amount of fourteen ounces, and one hundred (!) leeches were applied at two different times upon the course of the affected nerve. No permanent relief was obtained. Blisters were applied to the great trochanter and the head of the fibula, but the suffering of the patient appeared to be augmented.

February 7th.—Twenty-four drops of oil of turpentine were given in a julep, and continued each day until the 14th, and the disease was completely cured. From the 10th, the patient was quite relieved from pain, and her general health was much improved. Neither the stomach nor bowels suffered from the use of the remedy.

